# Supplementary material for: Human anogenital monocyte-derived dendritic cells and langerin+cDC2 are major HIV target cells
Source: Nat Commun. 2021 Apr 12;12:2147. doi: 10.1038/s41467-021-22375-x (PMC8042121; doi:10.1038/s41467-021-22375-x)
Supplement: Supplementary file 1 — Supplementary Information [file 41467_2021_22375_MOESM1_ESM.pdf]

Supplementary Figure 1

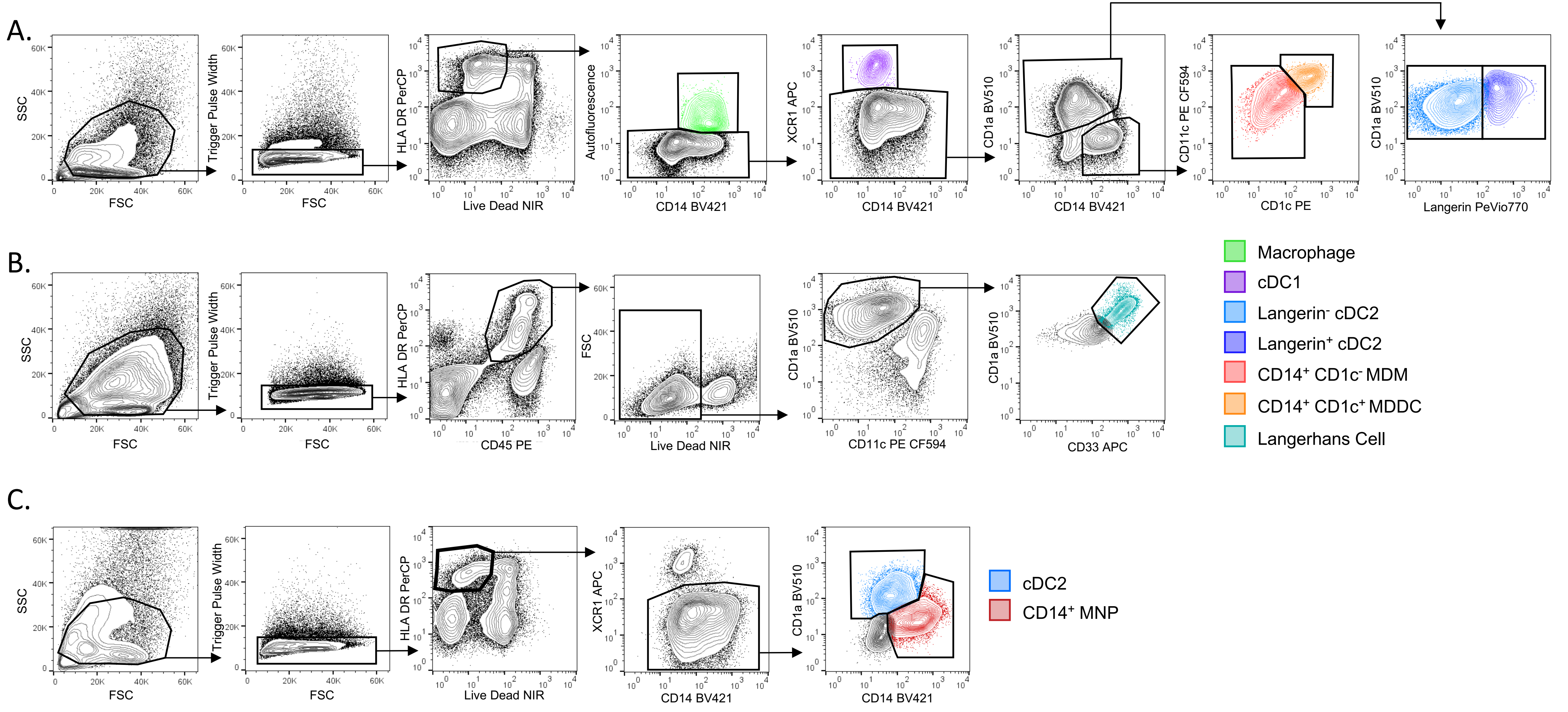

Supplementary Figure 1. Gating strategies for FACS sorting. **A.** Gating strategy to sort sub epithelial mononuclear phagocyte subsets including CD14^{+} autofluorescent macrophages (green), XCR1^{+} CD14^{-} cDC1 (purple), CD14^{+} CD1c^{-} MDM (red), CD14^{+} CD1c^{+} MDDC (orange), langerin^{-} cDC2 (light blue), langerin^{+} cDC2 (dark blue). Gating was used for RNAseq and HIV functional assays in figures 3A/B/D, 4A/C/D, 6A/B, 7C/E, 8C and 9C. **B.** Gating strategy to sort abdominal skin epidermal CD1a^{+} CD11c^{-} Langerhans cells (teal). Gating was used for RNAseq in figures 3A/D. **C.** Gating strategy for sub epithelial XCR1^{-} CD14^{-} CD1a^{+} cDC2 (blue) and XCR1^{-} CD1a^{-} CD14^{+} MNP (maroon). Gating was used for HIV functional assays in figures 6B, 7D and 9D. HLA DR^{+} Live cells (bold gate) were sorted for HIV uptake assays in figures 5A/C/E and 8A.

Supplementary table 1. RNAseq Primers	
oligo-dT Primer	5'-AAGCAGTGGTATCAACGCAGAGTACT$_{30}$VN-3'
TSO Primer	5'-AAGCAGTGGTATCAACGCAGAGTACATrGrG+G-3'
ISPCR Primer	5'-AAGCAGTGGTATCAACGCAGAGT-3'
