# Supplementary material for: Human anogenital monocyte-derived dendritic cells and langerin+cDC2 are major HIV target cells
Source: Nat Commun. 2021 Apr 12;12:2147. doi: 10.1038/s41467-021-22375-x (PMC8042121; doi:10.1038/s41467-021-22375-x)
Supplement: Supplementary file 3 — Reporting Summary [file 41467_2021_22375_MOESM3_ESM.pdf]

nature research

Corresponding author(s): Andrew Harman
Last updated by author(s): 05/03/2021

Reporting Summary

Nature Research wishes to improve the reproducibility of the work that we publish. This form provides structure for consistency and transparency in reporting. For further information on Nature Research policies, see our Editorial Policies and the Editorial Policy Checklist.

Statistics

For all statistical analyses, confirm that the following items are present in the figure legend, table legend, main text, or Methods section.

n/a	Confirmed	
☐	☒	The exact sample size (*n*) for each experimental group/condition, given as a discrete number and unit of measurement
☐	☒	A statement on whether measurements were taken from distinct samples or whether the same sample was measured repeatedly
☐	☒	The statistical test(s) used AND whether they are one- or two-sided<br>*Only common tests should be described solely by name; describe more complex techniques in the Methods section.*
☒	☐	A description of all covariates tested
☐	☒	A description of any assumptions or corrections, such as tests of normality and adjustment for multiple comparisons
☐	☒	A full description of the statistical parameters including central tendency (e.g. means) or other basic estimates (e.g. regression coefficient) AND variation (e.g. standard deviation) or associated estimates of uncertainty (e.g. confidence intervals)
☐	☒	For null hypothesis testing, the test statistic (e.g. *F*, *t*, *r*) with confidence intervals, effect sizes, degrees of freedom and *P* value noted<br>*Give P values as exact values whenever suitable.*
☒	☐	For Bayesian analysis, information on the choice of priors and Markov chain Monte Carlo settings
☒	☐	For hierarchical and complex designs, identification of the appropriate level for tests and full reporting of outcomes
☒	☐	Estimates of effect sizes (e.g. Cohen's *d*, Pearson's *r*), indicating how they were calculated

Our web collection on statistics for biologists contains articles on many of the points above.

Software and code

Policy information about availability of computer code

Data collection	Flow Cytometry data was collected using BD DIVA software (version 8.0). TZMBL results were collected using AID ELISPOT reader. Microscopy images were visualised using Olympus-ASW (version 2.9).
Data analysis	Flow cytometry data was analysed using FlowJo (V10). Microscopy analysis was done using OlyVia (version 2.9), FIJI (Madison version 2.0) and Huygens professional (20.04). All graphs/statistical analysis were generated using Prism- Graph pad (version 8.4.3). RNAseq data was analysed using TopHat2 software package (Version 2.1.1) and SAMMate (version 2.6.1)

For manuscripts utilizing custom algorithms or software that are central to the research but not yet described in published literature, software must be made available to editors and reviewers. We strongly encourage code deposition in a community repository (e.g. GitHub). See the Nature Research guidelines for submitting code & software for further information.

Data

Policy information about availability of data

All manuscripts must include a data availability statement. This statement should provide the following information, where applicable:

- Accession codes, unique identifiers, or web links for publicly available datasets
- A list of figures that have associated raw data
- A description of any restrictions on data availability

RNAseq data has been uploaded in the Gene Expression Omnibus (GEO) database under accession code GSE166639. Data availability statement is available in the manuscript Source data for all figures is available in the Source Data File.

April 2020

Field-specific reporting

Please select the one below that is the best fit for your research. If you are not sure, read the appropriate sections before making your selection.

☒ Life sciences ☐ Behavioural & social sciences ☐ Ecological, evolutionary & environmental sciences

For a reference copy of the document with all sections, see nature.com/documents/nr-reporting-summary-flat.pdf

Life sciences study design

All studies must disclose on these points even when the disclosure is negative.

Sample size	Sample sizes were not predetermined. For all key experiments at least three individual donors were analysed, while additional donors were used where ever possible. Due to low cell numbers in certain subsets (particularly langerin+ cDC2) it was not feasible to do some assays in larger numbers, however in each experiment we showed consistent trends in our results (which often reached statistical significance) and therefore believe sample size was sufficient.
Data exclusions	No data were excluded from the analysis
Replication	All findings presented were reproducible. For all functional assays findings were repeated at least three times with consistent results. For quantifying proportion of the mononuclear phagocyte subsets in human tissue there were a range of replications due to the varying commonality of receiving different tissues, with minimum replicates of 3 up to 52.
Randomization	There was no randomization in this study as we did not have different treatment/experimental groups and therefore was not relevant.
Blinding	There was no blinding in this study as we did not have different treatment/experimental groups and therefore was not relevant.

Reporting for specific materials, systems and methods

We require information from authors about some types of materials, experimental systems and methods used in many studies. Here, indicate whether each material, system or method listed is relevant to your study. If you are not sure if a list item applies to your research, read the appropriate section before selecting a response.

Materials & experimental systems

n/a	Involved in the study	
☐	☒	Antibodies
☐	☒	Eukaryotic cell lines
☒	☐	Palaeontology and archaeology
☒	☐	Animals and other organisms
☐	☒	Human research participants
☒	☐	Clinical data
☒	☐	Dual use research of concern

Methods

n/a	Involved in the study	
☒	☐	ChIP-seq
☐	☒	Flow cytometry
☒	☐	MRI-based neuroimaging

Antibodies

Antibodies used

Company: BD. Ab: CD103. Fluorphore: PE. Clone: Per-ACT8. Catalogue #: 340998 Lot #: 7250686
Company: BD. Ab: CD11c. Fluorphore: PECF594. Clone: B-Ly6. Catalogue #: 562393 Lot #: 9336670
Company: BD. Ab: CD14. Fluorphore: BUV737. Clone: M5E2. Catalogue #: 612763 Lot #: 140604
Company: Miltenyi. Ab: CD14. Fluorphore: Vioblue. Clone: TUK4. Catalogue #: 130-113-152.
Company: BD. Ab: CD14. Fluorphore: BV421. Clone: M5E2 Catalogue #: 565283 Lot #: 0.0064424
Company: BD. Ab: CD141. Fluorphore: BV711. Clone: 1A4. Catalogue #: 563155 Lot #: 9213223
Company: Miltenyi. Ab: CD141. Fluorphore: APC. Clone: AD5-14h12. Catalogue #: 130-113-314. Lot #: 5190605428
Company: BD. Ab: CD161(CLEC5b). Fluorphore: APC. Clone: DX12. Catalogue #: 550968 Lot #: 3291608
Company: Biolegend. Ab: CD169 (Siglec-1). Fluorphore: PE. Clone: 7-239. Catalogue #: 346004 Lot #: B223208
Company: eBioscience. Ab: CD172a (SIPRa). Fluorphore: PerCP-eFluor710. Clone: 15-414. Catalogue #: 46-1729-42
Company: Biolegend. Ab: CD172a (SIPRa). Fluorphore: APC/FIRE 750. Clone: SE5A5 Catalogue #: 323818 Lot #: B240662
Company: BD. Ab: CD184 (CXCR4). Fluorphore: PE. Clone: 12G5. Catalogue #: 555974 Lot #: 5260751
Company: Miltenyi. Ab: CD195 (CCR5). Fluorphore: PE. Clone: REA245. Catalogue #: 130-117-356. Lot #: 5200108299
Company: BD. Ab: CD1a. Fluorphore: BV510. Clone: HI149. Catalogue #: 563481 Lot #: 9109913
Company: BD. Ab: CD1c. Fluorphore: PE. Clone: F10/21A3 Catalogue #: 564900 Lot #: 8243815
Company: Miltenyi. Ab: CD1c. Fluorphore: PE-Vio770. Clone: AD5-8E7. Catalogue #: 130-113-865. Lot #: 5190626261
Company: Beckman Coulter. Ab: CD206 (MR). Fluorphore: PE. Clone: 3.29B1.10. Catalogue #: IM2741. Lot #: 15
Company: BD. Ab: CD206 (MR). Fluorphore: BUV805. Clone: 19.2. Catalogue #: 742042 Lot #: 9154863

April 2020

Company: Miltenyi. Ab: CD207 (Langerin). Fluorphore: Vioblue. Clone: MB22-9F2. Catalogue #: 130-106-096. Lot #: 5191016216
Company: Beckman Coulter. Ab: CD207 (Langerin). Fluorphore: PE. Clone: DCGM4. Catalogue #: IM3577.
Company: BD. Ab: CD209 (DC-SIGN). Fluorphore: APC. Clone: DCN46. Catalogue #: 551545
Company: R&D. Ab: CD299 (L-SIGN). Fluorphore: PE. Clone: 120604 Catalogue #: FAB162P. Lot #: LDR0311061
Company: R&D. Ab: CD301 (CLEC10a). Fluorphore: PE. Clone: 744812 Catalogue #: FAB48881P Lot #: ACTQ0112011
Company: BD. Ab: CD33 (Siglec-3). Fluorphore: APC. Clone: WM53. Catalogue #: 551378 Lot #: 5247669
Company: R&D. Ab: CD367 (CLEC4a). Fluorphore: PE. Clone: 216110 Catalogue #: FAB1748P. Lot #: LGQ0213041
Company: R&D. Ab: CD368 (CLEC4d). Fluorphore: PE. Clone: 413512 Catalogue #: FAB2806P. Lot #: ABJQ0113031
Company: BD. Ab: CD371 (CLEC12a). Fluorphore: AF674. Clone: 50C1. Catalogue #: 562568 Lot #: 7047639
Company: BD. Ab: CD4. Fluorphore: APC. Clone: RPA-T4. Catalogue #: 555349 Lot #: 3303853
Company: BD. Ab: CD45. Fluorphore: BV786. Clone: HI30. Catalogue #: 563716 Lot #: 813700
Company: BD. Ab: CD45. Fluorphore: PE. Clone: HI30 Catalogue #: 555483 Lot #: 7096274
Company: BD. Ab: CD80. Fluorphore: PE. Clone: L307.4. Catalogue #: 557227
Company: BD. Ab: CD83. Fluorphore: APC. Clone: HB15e. Catalogue #: 551073 Lot #: 9009957
Company: BD. Ab: CD86. Fluorphore: APC. Clone: 2331 Catalogue #: 555660 Lot #: 8235916
Company: eBioscience. Ab: CD91. Fluorphore: eFluor660. Clone: A2MR-a2. Catalogue #: 50-0919-41 Lot #: E24664-101
Company: R&D. Ab: CLEC14a. Fluorphore: APC. Clone: 743940 Catalogue #: FAB7436A Lot #: ACUQ0112021
Company: R&D. Ab: CLEC5a. Fluorphore: APC. Clone: 283834 Catalogue #: FAB2384A Lot #: abju0115081
Company: R&D. Ab: CLEC5C. Fluorphore: APC. Clone: 239127 Catalogue #: FAB1900A-100 Lot #: LSS0313121
Company: R&D. Ab: CLEC6a (Dectin2). Fluorphore: APC. Clone: 545943 Catalogue #: FAB3114P Lot #: ABFE0213101
Company: Miltenyi. Ab: CLEC7A (DECTIN1). Fluorphore: PE. Clone: REA515. Catalogue #: 130-122-009 Lot #: 5160406364
Company: Miltenyi. Ab: CLEC9A (CD370). Fluorphore: PE. Clone: 8F9. Catalogue #: 130-099-615 Lot #: 5141210153
Company: BD. Ab: DC SIGN. Fluorphore: PE. Clone: DCN46 Catalogue #: 551265 Lot #: 2097966
Company: Biolegend. Ab: DEC205. Fluorphore: PE. Clone: HD30. Catalogue #: 342204 Lot #: B159801
Company: Miltenyi. Ab: HLA DR. Fluorphore: PerCP. Clone: AC122. Catalogue #: 130-113-966 Lot #: 5200108204
Company: BD. Ab: HLA-DR. Fluorphore: BUV395. Clone: G46-6. Catalogue #: 564040 Lot #: 9310
Company: Miltenyi. Ab: Langerin. Fluorphore: PE-Vio770. Clone: MB22-9F2. Catalogue #: 130-100-586 Lot #: 5191023228
Company: BD. Ab: Mouse IgG1. Fluorphore: APC. Clone: MOPC-21. Catalogue #: 555751 Lot #: 8081995
Company: BD. Ab: Mouse IgG1. Fluorphore: PE. Clone: MOPC-21C. Catalogue #: 550617 Lot #: 2160609
Company: Miltenyi. Ab: Mouse IgG1. Fluorphore: APC. Clone: IS5-21F5. Catalogue #: 130-113-200.
Company: Beckman Coulter. Ab: Mouse IgG1. Fluorphore: PE. Clone: 679.1Mc7. Catalogue #: IM0670U
Company: ebioscience. Ab: Mouse IgG1. Fluorphore: eFluor660. Clone: P3.6.2.8.1. Catalogue #: 50-4714-80. Lot #: E12137-1634.
Company: R&D. Ab: Mouse IgG1. Fluorphore: PE. Clone: 11711 Catalogue #: IC002P. Lot #: LGZ2913031
Company: BD. Ab: Mouse IgG2a. Fluorphore: PE. Clone: ZERO8894. Catalogue #: 555574 Lot #: 0.008894
Company: Miltenyi. Ab: Mouse IgG2a. Fluorphore: PE. Clone: 5140124038 Catalogue #: 130-091-835. Lot #: 5140124038
Company: R&D. Ab: Mouse IgG2a. Fluorphore: APC. Clone: 20102 Catalogue #: IC003A Lot #: LHA1013111
Company: R&D. Ab: Mouse IgG2b. Fluorphore: APC. Clone: 133303 Catalogue #: IC0041A Lot #: II011041
Company: BD. Ab: Mouse IgG2b. Fluorphore: APC. Clone: 27-35 Catalogue #: 555745 Lot #: 36953
Company: Beckman Coulter. Ab: P24. Fluorphore: RDI. Clone: KC57. Catalogue #: 6604667 Lot #: 7433056
Company: MedimAb. Ab: P24. Fluorphore: APC. Clone: 28B7. Catalogue #: MM-0289-APC Lot #: 28902029113
Company: Merck. Ab: SAMHD1. Fluorphore: Unconjugated Clone: I-19-18. Catalogue #: MABF933 Lot #: 3172409
Company: EBioscience. Ab: SIGLEC1. Fluorphore: PE eFluor610. Clone: 7-239. Catalogue #: 61-1699-42 Lot #: 2105871
Company: R&D. Ab: Siglec16. Fluorphore: APC. Clone: 706022 Catalogue #: FAB6819A Lot #: ACFR0111061
Company: R&D. Ab: Siglec9. Fluorphore: APC. Clone: 191240 Catalogue #: FAB1139A-100 Lot #: AACT0214011
Company: Biolegend. Ab: XCR1. Fluorphore: APC. Clone: S15046E. Catalogue #: 372606 Lot #: B300821.
Company: R&D. Host species: Goat. Ab: Langerin. Clone: Polyclonal Catalogue #: AF2088. Lot #: KQJD115051.
Company: abcam. Host species: Rabbit. Ab: CD14. Clone: ERP3652. Catalogue #: ab133503 Lot #: GR211954-4.
Company: abcam. Host species: Rabbit. Ab: CD11c. Clone: EP1347Y. Catalogue #: ab216655
Company: Invitrogen. Host species: Donkey. Ab: anti goat. Fluorphore: AF488. Catalogue #: A11055. Lot #: 49732A.
Company: Invitrogen. Host species: Donkey. Ab: anti rabbit. Fluorphore: DyLight755. Catalogue #: SA5-10043. Lot #: TA2506986.

Validation

All antibodies used were commercially available and validated by corresponding manufacturers. The following companies have general validation/reproducibility statements. BD (https://www.bdbiosciences.com/us/go/reproducibility/overview). Biolegend (https://www.biolegend.com/en-us/reproducibility). Beckman Coulter (https://www.beckman.com.au/reagents/coulter-flow-cytometry/antibodies-and-kits/single-color-antibodies).

CD103. PE. https://www.bdbiosciences.com/us/applications/research/t-cell-immunology/regulatory-t-cells/surface-markers/human/pe-mouse-anti-human-cd103-ber-act8/p/340998

CD11c. PECF594. https://www.bdbiosciences.com/us/reagents/research/antibodies-buffers/immunology-reagents/anti-human-antibodies/cell-surface-antigens/pe-cf594-mouse-anti-human-cd11c-b-ly6/p/562393

CD14. BUV737. https://www.bdbiosciences.com/us/reagents/research/antibodies-buffers/immunology-reagents/anti-human-antibodies/cell-surface-antigens/buv737-mouse-anti-human-cd14-m5e2/p/612763

CD14. Vioblue. https://www.miltenyibiotec.com/AU-en/products/cd14-antibody-anti-human-tuk4.html?countryRedirected=1#vioblue:100-tests-in-200-ul

CD14. BV421. https://www.bdbiosciences.com/us/reagents/research/antibodies-buffers/immunology-reagents/anti-human-

antibodies/cell-surface-antigens/bv421-mouse-anti-human-cd14-m5e2/p/565283

CD141. BV711. https://www.bdbiosciences.com/us/applications/research/stem-cell-research/mesoderm-markers/human/bv711-mouse-anti-human-cd141-1a4/p/563155

CD141. APC. https://www.miltenyibiotec.com/AU-en/products/cd141-bdca-3-antibody-anti-human-ad5-14h12.html#gref

CD161(CLEC5b). APC. https://www.bdbiosciences.com/eu/reagents/research/antibodies-buffers/immunology-reagents/anti-human-antibodies/cell-surface-antigens/apc-mouse-anti-human-cd161-dx12/p/550968

CD169 (Siglec-1). PE. https://www.biolegend.com/en-us/products/pe-anti-human-cd169-sialoadhesin-siglec-1-antibody-6290

CD172a (SIPRa). PerCP-eFluor710. https://www.thermofisher.com/antibody/product/CD172a-SIRP-alpha-Antibody-clone-15-414-Monoclonal/46-1729-42

CD172a (SIPRa). APC/FIRE 750. https://www.biolegend.com/en-us/products/apc-fire-750-anti-human-cd172ab-sirpalphabeta-antibody-14802

CD184 (CXCR4). PE. https://www.bdbiosciences.com/eu/applications/research/t-cell-immunology/regulatory-t-cells/surface-markers/human/pe-mouse-anti-human-cd184-12g5/p/555974

CD195 (CCR5). PE. https://www.miltenyibiotec.com/AU-en/products/cd195-ccr5-antibody-anti-human-reafinity-rea245.html#gref

CD1a. BV510. https://www.bdbiosciences.com/us/reagents/research/antibodies-buffers/immunology-reagents/anti-human-antibodies/cell-surface-antigens/bv510-mouse-anti-human-cd1a-hi149/p/563481

CD1c. PE. https://www.bdbiosciences.com/eu/reagents/research/antibodies-buffers/immunology-reagents/anti-human-antibodies/cell-surface-antigens/pe-mouse-anti-human-cd1c-f1021a3/p/564900

CD1c. PE-Vio770. https://www.miltenyibiotec.com/AU-en/products/cd1c-bdca-1-antibody-anti-human-ad5-8e7.html#gref

CD206 (MR). PE. https://www.beckman.com.au/reagents/coulter-flow-cytometry/antibodies-and-kits/single-color-antibodies/cd206/im2741

CD206 (MR). BUV805. https://www.bdbiosciences.com/us/reagents/research/antibodies-buffers/immunology-reagents/anti-human-antibodies/cell-surface-antigens/buv805-mouse-anti-human-cd206-192/p/742042

CD207 (Langerin). Vioblue. https://www.miltenyibiotec.com/AU-en/products/cd207-langerin-antibody-anti-human-mb22-9f5.html?countryRedirected=1#gref

CD207 (Langerin). PE. https://www.beckman.com.au/reagents/coulter-flow-cytometry/antibodies-and-kits/single-color-antibodies/cd207/im3577

CD209 (DC-SIGN). APC. https://www.bdbiosciences.com/us/applications/research/stem-cell-research/mesoderm-markers/human/apc-mouse-anti-human-cd209-dcn46/p/551545

CD299 (L-SIGN). PE. https://www.rndsystems.com/products/human-dc-signr-cd299-pe-conjugated-antibody-120604_fab162p

CD301 (CLEC10a). PE. https://www.rndsystems.com/products/human-clec10a-cd301-pe-conjugated-antibody-744812_fab48881p

CD33 (Siglec-3). APC. https://www.bdbiosciences.com/eu/applications/research/stem-cell-research/hematopoietic-stem-cell-markers/human/negative-markers/apc-mouse-anti-human-cd33-wm53-also-known-as-wm-53/p/551378

CD367 (CLEC4a). PE. https://www.rndsystems.com/products/human-dcir-clec4a-pe-conjugated-antibody-216110_fab1748p

CD368 (CLEC4d). PE. https://www.rndsystems.com/products/human-clec4d-clecsf8-pe-conjugated-antibody-413512_fab2806p

CD371 (CLEC12a). AF674. https://www.bdbiosciences.com/us/reagents/research/antibodies-buffers/immunology-reagents/anti-human-antibodies/cell-surface-antigens/alexa-fluor-647-mouse-anti-human-cd371-clec12a-50c1/p/562568

CD4. APC. https://www.bdbiosciences.com/eu/applications/research/t-cell-immunology/th-1-cells/surface-markers/human/apc-mouse-anti-human-cd4-rpa-t4/p/555349

CD45. BV786. https://www.bdbiosciences.com/us/applications/research/stem-cell-research/cancer-research/human/bv786-mouse-anti-human-cd45-hi30/p/563716

CD45. PE. https://www.bdbiosciences.com/eu/applications/research/stem-cell-research/cancer-research/human/pe-mouse-anti-human-cd45-hi30/p/555483

CD80. PE. https://www.bdbiosciences.com/eu/applications/research/b-cell-research/surface-markers/human/pe-mouse-anti-human-

cd80-l3074-also-known-as-l307/p/557227

CD83. APC. https://www.bdbiosciences.com/us/reagents/research/antibodies-buffers/immunology-reagents/anti-human-antibodies/cell-surface-antigens/apc-mouse-anti-human-cd83-hb15e/p/551073

CD86. APC. https://www.bdbiosciences.com/eu/applications/research/t-cell-immunology/regulatory-t-cells/surface-markers/human/apc-mouse-anti-human-cd86-2331-fun-1/p/555660

CD91. eFluor660. https://www.thermofisher.com/order/genome-database/dataSheetPdf?producttype=antibody&productsubtype=antibody_primary&productId=%2050-0919-41

CLEC14a. APC. https://www.rndsystems.com/products/human-clec14a-apc-conjugated-antibody-743940_fab7436a

CLEC5a. APC. https://www.rndsystems.com/products/human-mdl-1-clec5a-apc-conjugated-antibody-283834_fab2384a

CLEC5C. APC. https://www.rndsystems.com/products/human-nkp80-klrf1-apc-conjugated-antibody-239127_fab1900a

CLEC6a (Dectin2). APC. https://www.rndsystems.com/products/human-dectin-2-clec6a-pe-conjugated-antibody-545943_fab3114p

CLEC7A (DECTIN1). PE. https://www.miltenyibiotec.com/AU-en/products/dectin-1-antibody-anti-human-reafinity-rea515.html?countryRedirected=1#pe:30-tests-in-60-ul

CLEC9A (CD370). PE. https://www.miltenyibiotec.com/AU-en/products/cd370-clec9a-antibody-anti-human-8f9.html#gref

DC SIGN. PE. https://www.bdbiosciences.com/us/applications/research/stem-cell-research/mesoderm-markers/human/pe-mouse-anti-human-cd209-dcn46/p/551265

DEC205. PE. https://www.biolegend.com/en-us/products/pe-anti-human-cd205-dec-205-antibody-5901?GroupID=GROUP28

HLA DR. PerCP. https://www.miltenyibiotec.com/AU-en/products/hla-dr-antibody-anti-human-ac122.html?countryRedirected=1#gref

HLA-DR. BUV395. https://www.bdbiosciences.com/us/applications/research/stem-cell-research/mesenchymal-stem-cell-markers-bone-marrow/human/negative-markers/buv395-mouse-anti-human-hla-dr-g46-6/p/564040

Langerin. PE-Vio770. https://www.miltenyibiotec.com/AU-en/products/cd207-langerin-antibody-anti-human-mb22-9f5.html?countryRedirected=1#pe-vio-770:100-tests-in-1-ml

Mouse IgG1. APC. https://www.bdbiosciences.com/eu/reagents/research/antibodies-buffers/immunology-reagents/anti-human-antibodies/cell-surface-antigens/apc-mouse-igg1-isotype-control-mopc-21/p/555751

Mouse IgG1. PE. https://www.bdbiosciences.com/us/reagents/research/antibodies-buffers/immunology-reagents/anti-mouse-antibodies/cell-surface-antigens/pe-mouse-igg1-isotype-control-mopc-31c/p/550617

Mouse IgG1. APC. https://www.miltenyibiotec.com/AU-en/products/isotype-control-antibody-mouse-igg1-is5-21f5.html#apc:30-tests-in-60-ul

Mouse IgG1. PE. https://www.beckman.com.au/reagents/coulter-flow-cytometry/antibodies-and-kits/single-color-antibodies/isotype-control/im0670u

Mouse IgG1. eFluor660. https://www.fishersci.co.uk/shop/products/igg1-k-mouse-efluor-660-clone-p3-6-2-8-1-isotype-control-ebioscience-2/15321910

Mouse IgG1. PE. https://www.rndsystems.com/products/mouse-igg-1-pe-conjugated-antibody_ic002p

Mouse IgG2a. PE. https://www.bdbiosciences.com/us/reagents/research/antibodies-buffers/immunology-reagents/anti-human-antibodies/cell-surface-antigens/pe-mouse-igg2a-isotype-control-g155-178/p/555574

Mouse IgG2a. PE. https://www.citeab.com/antibodies/2090934-130-091-835-mouse-igg2a-pe-monoclonal

Mouse IgG2a. APC. https://www.rndsystems.com/products/mouse-igg2a-apc-conjugated-antibody_ic003a

Mouse IgG2b. APC. https://www.rndsystems.com/products/mouse-igg2b-apc-conjugated-antibody_ic0041a

Mouse IgG2b. APC. https://www.bdbiosciences.com/us/reagents/research/antibodies-buffers/immunology-reagents/anti-human-antibodies/cell-surface-antigens/apc-mouse-igg2b-isotype-control-27-35/p/555745

P24. RDI. https://www.beckman.com.au/reagents/coulter-flow-cytometry/antibodies-and-kits/single-color-antibodies/hiv-1-core-antigen/6604667

P24. APC. https://medimabs.com/product/hiv-1-p24-human-monoclonal-antibody-28b7-apc-labeled/

SAMHD1. Unconjugated https://www.merckmillipore.com/AU/en/product/Anti-SAMHD1-Antibody-clone-I19-18,MM_NF-MABF933

SIGLEC1. PE eFluor610. https://www.fishersci.co.uk/shop/products/cd169-siglec-1-mouse-anti-human-pe-efluor-610-clone-7-239-ebioscience-2/15580467

Siglec16. APC. https://www.rndsystems.com/products/human-siglec-16-apc-conjugated-antibody-706022_fab6819a

Siglec9. APC. https://www.rndsystems.com/products/human-siglec-9-apc-conjugated-antibody-191240_fab1139a

XCR1. APC. https://www.biolegend.com/en-us/search-results/apc-anti-human-xcr1-antibody-14678

Goat Langerin. https://www.rndsystems.com/products/human-langerin-cd207-antibody_af2088

Rabbit CD14. https://www.abcam.com/cd14-antibody-epr3652-ab133503.html

Rabbit CD11c. https://www.abcam.com/cd11c-antibody-ep1347y-bsa-and-azide-free-ab216655.html

Donkey anti goat AF488 https://www.thermofisher.com/antibody/product/Donkey-anti-Goat-IgG-H-L-Cross-Adsorbed-Secondary-Antibody-Polyclonal/A-11055

Donkey anti rabbit DyLight755 https://www.thermofisher.com/antibody/product/Donkey-anti-Rabbit-IgG-H-L-Cross-Adsorbed-Secondary-Antibody-Polyclonal/SA5-10043

Eukaryotic cell lines

Policy information about cell lines

Cell line source(s)	JLTR - NIH AIDS repository (Cat #11586)<br>TZMBL1 - NIH AIDS repository (Cat # 100)
Authentication	Cell lines are both reporter cell lines which will only report (Fluoresce green or turn blue) when infected with HIV. Furthermore STR profiling was conducted on both cell lines.
Mycoplasma contamination	Mycoplasma testing was regularly performed and cells lines were shown to be mycoplasma free
Commonly misidentified lines (See ICLAC register)	Neither cell line are listed in the most recent ICLAC database of commonly misidentified cell lines.

Human research participants

Policy information about studies involving human research participants

Population characteristics	Healthy human abdominal and genital tissues were obtained within 30 minutes of surgery from patients undergoing plastic surgery (Abdomen, labia and vagina), circumcision (foreskin), gender reassignment (glans penis, fossa navicularis, penile urethra), hysterectomy (cervix) or colorectal surgery (anal canal, perineum, colon, rectum). All patients were aged between 16-71 and gave written consent. All tissues were de-identified.
Recruitment	Human tissue was collected from a range of different surgeons across Sydney whom are named as authors in this study. There were no self-selection biases, any individual who was undergoing relevant surgery was asked at consult if they would like to donate their discarded tissue to the study and then consented to the study. There were no specific requirements for patients to meet for tissue to be included in this study, however macroscopically "diseased" tissue was sent to pathology and therefore omitted from this study.
Ethics oversight	The study was approved by the Western Sydney Local Health District (WSHLD) Human Research Ethics Committee (HREC); reference number (4192) AU RED HREC/15 WMEAD/11.

Note that full information on the approval of the study protocol must also be provided in the manuscript.

Flow Cytometry

Plots

Confirm that:

- [x] The axis labels state the marker and fluorochrome used (e.g. CD4-FITC).
- [x] The axis scales are clearly visible. Include numbers along axes only for bottom left plot of group (a 'group' is an analysis of identical markers).
- [x] All plots are contour plots with outliers or pseudocolor plots.
- [x] A numerical value for number of cells or percentage (with statistics) is provided.

Methodology

Sample preparation	Cells were isolated from human anogenital and colorectal tissue as well as abdominal skin using enzymatic digestions. For abdominal skin, grafts were taken using a skin graft knife and passed through a skin graft mesher. For anogential skin and type II mucosasl tissue, underlying fat/connective tissue was removed with a scalpel before small scalpel cuts were made to the epithelial surface to mimick that of the skin graft mesher. Tissue were incubated overnight at 4˚C with 0.14U/ml dispase in RPMI. Skin/type II mucosa was briefly washed in PBS and epidermis and dermis mechanically seperated using forceps and cut into 1-2mm pieces for digestion in collagenase type IV (200U/ml) with DNase1 (100U/ml) in RPMI at 37˚C for two hours. Undigested tissue was removed using a tea strainer and supernatant than passed through a 100µm cell strainer and pelleted three times in PBS. Epidermal suspension underwent Ficoll-Paque enrichment while dermal suspensions were enriched using CD45 MACS bead selection. For type I mucosa no dispase treatment was required, tissue was digested in type IV collagenase over two consecutive 30 minute digestions.<br>Tissues included in this study were as follows -<br>Skin: Abdomen, Labia, Glans, Inner and outer foreskin, Perinuem.<br>Type II mucosa: Vagina, Fossa navicularis, Anal Canal, Ectocervix<br>Type I mucosa: Endocervix, Penile urethra, Rectum, Colon
Instrument	BD fortessa, BD CantoII (transfer assays only), BD Symphony (Siglec-1 blocking experiment only) BD influx (FACS)
Software	Collected using BD DIVA (V 8.0) and analysed using FlowJo (V10).
Cell population abundance	All sorted populations were purity checked by running post-sorted samples back through the sorter and checking their surface expression. Purity checks were 99% accurate. Cell abundance varied with tissue source and size as well as cell subset from 5000-500000 sorted cells
Gating strategy	All antibodies were titrated on PBMCs and primary abdominal skin cells to determine optimal concentrations for separation of negative and positive cells.<br>FSC/SSC to gate broadly on immune cells. CD45+ HLADR+ cells were gated, followed by excluding dead cells by Live/Dead viability dye. Doublets were excluded using FSC-A/FSC-H. T cells were excluded as CD3+ cells and B cells excluded as CD19+. From here populations were gated out as follows:<br>Macrophages: Autofluorescent+ CD14+<br>cDC1: Autofluorescent- CD14- XCR1+<br>langerin- cDC2: Autofluorescent- XCR1- CD14- CD1c+ (Mucosal tissue) CD1a+ (skin) Langerin-<br>langerin+ cDC2: Autofluorescent- XCR1- CD14- CD1c+ (Mucosal tissue) CD1a+ (skin) Langerin+<br>CD14+ MDDC: Autofluorescent- XCR1- CD14+ CD1a- (skin) CD1c+ CD11c+<br>CD14+ MDM: Autofluorescent- XCR1- CD14+ CD1a- (skin) CD1c- CD11c-<br>HIV+ cells were gated as p24 (KC57) RDI+ p24 (28B7) APC+.<br>All population markers were on bifurcated markers and clearly defined positive from negative. All CLRs/markers of interest were gated using FMOs or isotype controls.

- [x] Tick this box to confirm that a figure exemplifying the gating strategy is provided in the Supplementary Information.
